# On the Menu: Analyzing the Macronutrients, Micronutrients, Beverages, Dietary Patterns, and Pancreatic Cancer Risk

**DOI:** 10.7759/cureus.45259

**Published:** 2023-09-14

**Authors:** Sonia Mukhtar, Ali Moradi, Athri Kodali, Chiugo Okoye, Dhadon Klein, Iman Mohamoud, Olawale O Olanisa, Panah Parab, Priti Chaudhary, Pousette Hamid

**Affiliations:** 1 Internal Medicine, California Institute of Behavioral Neurosciences & Psychology, Fairfield, USA; 2 Medicine, Semmelweis University, Budapest, HUN; 3 Medicine, California Institute of Behavioral Neurosciences & Psychology, Fairfield, USA; 4 Neurology, California Institute of Behavioral Neurosciences & Psychology, Fairfield, USA

**Keywords:** risk of pancreatic cancer, mediterranean diet (md), pancreatic cancer, western diet, micronutrients, macronutrients, food and dietary patterns

## Abstract

This narrative review summarizes the principal findings of observational studies, systematic reviews, and meta-analyses on diet and dietary patterns' role in the risk of pancreatic cancer. Etiologically pancreatic cancer is multifactorial. Evidence exists of an association between nutrients, dietary patterns, and pancreatic cancer. An extensive literature search was conducted on PubMed, Cochrane, and Google Scholar. A thorough search of articles published in English till May 2023 and related to the review was performed. The relationship between all macronutrients, micronutrients, and various dietary patterns with the risk of pancreatic cancer was assessed. It is concluded that a diet high in nutrients like red and processed meat, refined sugars, saturated and monounsaturated fats, alcohol, copper, and a Western dietary pattern can increase the likelihood of pancreatic cancer. Contrary to this, a diet consisting of fruits, vegetables, appropriate quantities of vitamins and minerals, and a Mediterranean dietary pattern is associated with a decreased risk of pancreatic cancer.

## Introduction and background

There is proof of a relationship between dietary patterns and various malignancies, but it could be imprecise due to intrinsic biases. Alcohol consumption increases the risk of colorectal and breast cancer, whereas whole grain products and calcium intake decrease the risk of colorectal cancer [1,2]. 

Pancreatic cancer is the 12th most common cancer in the world and the seventh leading cause of cancer death in both males and females. With its dismal outlook, pancreatic cancer is projected to become the second leading cause of cancer-related deaths globally by 2040. It accounts for almost as many deaths (466,000) as cases (496,000) [3,4]. As the population ages, the incidence of pancreatic cancer will continue to increase. The disability-adjusted life year, incidence, and number of deaths have doubled globally from 1990 to 2017. The absence of disease-defining symptoms until the advanced stage of cancer, difficulty in early diagnosis, and ability to metastasize quickly are the major causes of high mortality [5,6]. Due to the unavailability of a practical approach and low incidence, no screening programs have been established for asymptomatic patients, despite early diagnosis being pivotal to survival rates [7]. Due to the lack of established screening methods, primary prevention by modifying the risk factors is likely the most efficient means of decreasing the burden of pancreatic cancer. 

Pancreatic cancer is a multifactorial disease etiologically, with pathogenesis including several genetic and environmental factors such as cystic fibrosis, hereditary breast and ovarian cancer syndrome, familial adenomatous polyposis, Peutz-Jeghers syndrome, hereditary pancreatitis, alcohol consumption, cigarette smoking, diabetes mellitus, obesity, and chronic pancreatitis [8]. 

Although diet is a significant modifiable lifestyle factor, epidemiological studies reported inconsistent results regarding the relationship between dietary intake or individual nutrients such as processed meat, red meat, vitamins and minerals, fibers, fat, vegetables, and fruits, and pancreatic cancer [9,10]. Many methods are available for categorizing dietary patterns in a population, but primarily data-driven and index-based approaches are used. There is little agreement on which type of dietary pattern analysis should be used for a particular objective, although no one approach is considered superior to all the others. The reproducibility and credibility of dietary assessment tools have been questioned and likely depend on the characteristics of measurement tools [11]. The sparsity of data on the relationship between patterns of dietary intake and pancreatic cancer prompted us to perform a literature review of the already published studies in an effort to consolidate the available data.

## Review

Macronutrients and pancreatic cancer

Carbohydrates

A study by Michaud et al. showed that a diet high in carbohydrates could increase the risk of pancreatic cancer, especially in overweight women with a sedentary lifestyle. During an 18-year follow-up period, 180 case subjects with pancreatic cancer were identified in a cohort of American women (n = 88,802). To calculate sucrose, fructose, and carbohydrate intake, the frequency of intake of individual foods, as reported on the food-frequency questionnaire, was used. The study showed that the glycemic load was directly related to carbohydrate intake and the glycemic index. In women with some degree of insulin resistance, a diet high in glycemic load may enhance their chance of developing pancreatic cancer [12].

A systematic review and meta-analysis of 10 cohort studies by D. Aune et al. that analyzed the relationship between glycemic load consumption, glycemic index, carbohydrate intake, and pancreatic cancer showed a relative risk of 1.04 [95% confidence interval (CI): 0.93-1.17] in high versus low analysis for pancreatic and gastrointestinal cancer risk associated with glycemic index, a relative risk of 1.01 [95% CI: 0.88-1.15]. Eight cohort studies included 2986 cases among 1,031,893 participants in the high versus low analysis of pancreatic cancer risk and glycemic index. The relative risk was 1.04, with a confidence interval of 0.93-1.17. Nine cohort studies included 3420 cases among 1,194,043 participants in the high versus low analysis of pancreatic cancer risk and glycemic index. The relative risk was 1.01, with a confidence interval of 0.88-1.15. Nine cohort studies included 3202 cases among 1,112,404 participants in the high versus low analysis of pancreatic cancer risk and total carbohydrate intake. The relative risk was 1.00, with a confidence interval of 0.86-1.15. The study concluded that increased fructose intake but not total carbohydrates or sucrose increases the risk of pancreatic cancer [13].

Several mechanisms have been proposed to explain the relationship between carbohydrate intake and the risk of pancreatic cancer. Increased insulin secretions and insulin resistance can occur from diets with a high glycemic index and glycemic load, which may promote the development of pancreatic cancer through their mitogenic and anti-apoptotic effects [14,15]. Chronic hyperglycemia and hyperinsulinemia can lead to uncontrolled insulin signaling, which can cause the multiplication and survival of pancreatic cells by activating various signaling pathways [16]. A diet high in refined carbohydrates and simple sugars can cause metabolic syndrome and obesity, risk factors for pancreatic cancer [17]. The relationship between a diet high in carbohydrates and pancreatic cancer is complicated, and the mechanisms involved are poorly understood, which warrants further studies. 

Dietary Proteins and Amino Acids

There is increasing evidence that proteins are involved in several biological processes as integral macronutrients and might play a role in the development and progression of pancreatic cancer. 

Regarding the consumption of animal protein, there is convincing evidence from several studies indicating the association of fresh and total red meat (including processed) intake with an increased risk of pancreatic cancer. A meta-analysis of 11 prospective studies analyzing 6643 pancreatic cancer patients showed a statistically significant positive association between increased consumption of fresh red meat, pork, or total red meat by 120 grams per day and an elevated risk of pancreatic cancer in men but no statistically significant association in women. The analysis showed a statistically significant increase in pancreatic cancer risk and increased consumption of processed meat by 50 grams daily [18]. Grilling and frying animal proteins has been linked to the production of carcinogenic compounds such as heterocyclic amines and polycyclic aromatic hydrocarbons, which may contribute to pancreatic cancer development. The amount of meat consumed is directly related to the increased risk of pancreatic cancer and is also dependent on age, with the major concern of risk in people with age above 60 [19]. 

Plant-based proteins like legumes, nuts, and whole grains have been associated with a reduced risk of pancreatic cancer at variance with meat proteins. A subgroup analysis of a recent study assessing the association between a plant-based diet and gastrointestinal cancer showed a statistically significant risk reduction with plant-based protein intake and pancreatic cancer, with a relative risk of 0.71 [95% CI: 0.59-0.86, P < 0.001] [20].

There could be multiple routes through which protein diets can influence pancreatic cancer. Consumption of animal protein might trigger the activation of the mammalian target of the rapamycin (mTOR) pathway, which is known to enhance cell growth and proliferation [21]. Additionally, animal proteins have the potential to stimulate insulin-like growth factor (IGF) signaling, which has been linked to the development of tumors [22]. However, additional prospective studies are necessary to investigate the precise connection between animal-based proteins and the likelihood of pancreatic cancer and the potential protective effect of plant-based proteins against this risk.

Dietary Fats and Fatty Acids

Several studies have investigated the relationship between dietary fat intake and an increased risk of pancreatic cancer, but the results have been inconsistent. A prospective cohort study analyzed a cohort of 308,736 men and 216,737 women for the association between fat, fat subtypes, and pancreatic cancer. Among the patients diagnosed with pancreatic cancer after more than six years of follow-up, a direct relationship was found between pancreatic cancer risk and intake of total fat, saturated fat, and monounsaturated fat, but no association was found with polyunsaturated fat. The most vital relationship was found between the intake of animal fat, including red meat, and dairy products in terms of increasing the risk of pancreatic cancer. The link between red meat consumption (high in saturated fat) and pancreatic cancer was found to have a marginally statistically significant sex-based interaction, with men displaying a strong statistically significant correlation while women showed no such link. On the other hand, both men and women were found to have a statistically significant positive association between dairy consumption of saturated fat and pancreatic cancer [23]. 

A case-controlled study conducted at the Mayo Clinic USA showed polyunsaturated fatty acids, beta-tocopherol, and linoleic acid had an inverse relationship with pancreatic cancer; in contrast, saturated fatty acids such as butyric acid, myristic acid, stearic acid, trans-octadecadienoic acid, caproic acid, caprylic acid, and capric acid had an increased risk of the disease. Cases were most likely to have a history of new-onset diabetes. The meta-analysis found no links between the intake of eggs, poultry, or fish and pancreatic cancer, and the studies that indicated associations between red meat consumption and increased risk were primarily case-control studies [24]. 

According to another study, the usage of fat in seasoning was judged using subjective scores (low, intermediate, high) for butter, margarine, and oil consumption. Butter and margarine showed no material correlation. However, the consumption of olive oil has been associated inversely with pancreatic cancer [25]. Many of the studies conducted in this area rely on self-reported dietary data, which can be subject to inaccuracies and biases. Moreover, pancreatic cancer is a multifactorial disease, and dietary factors alone are unlikely to be the sole cause of its development. Further prospective studies are required to explore an association between dietary fat and pancreatic cancer.

Micronutrients

Vitamins

Vitamin D:* *Epidemiological studies have shown inconsistent results regarding the association between pancreatic cancer and vitamin D concentrations. According to a nested case-control study of eight cohorts analyzing 952 patients with pancreatic cancer, no significant relationship was found between low vitamin D concentrations and pancreatic cancer. However, a twofold increase in pancreatic cancer was seen with a high concentration of vitamin D >100 nmol/L. Cases reported smoking more frequently, ingesting more saturated fat, and consuming less fish, carbohydrates, and folate than controls. There was no statistically significant difference between the proportions of patients and controls with vitamin D values less than 37.5 nmol/L or less than 25 nmol/L [26]. In a recent dose-response meta-analysis, daily consumption of 10 μg/d vitamin D was found to reduce the risk of pancreatic cancer by 25% [27]. However, the evidence is inconclusive, and more research is needed to establish a definitive relationship. It is important to note that while these findings are intriguing, they do not provide definitive proof of causation or establish vitamin D as a preventive or therapeutic agent for pancreatic cancer. 

Vitamin E:* *Several epidemiological studies have suggested a reduction in pancreatic cancer risk with vitamin E intake. One of the studies analyzed 10 observational studies, including six case-control studies and four cohort studies with 2976 patients and 254,393 participants, respectively. The populations of four studies were from the USA, two were from Asia (Japanese and Chinese), and four were from Europe. These studies found an inverse correlation between vitamin E intake and pancreatic cancer. In these studies, there was little statistically significant evidence of heterogeneity [28]. Vitamin E has been demonstrated to be a beneficial antioxidant since oxidation has been connected to a wide range of potential ailments and diseases, including cancer, aging, arthritis, and cataracts. Vitamin E is known for its antioxidant properties, which help protect cells from oxidative damage [29]. Pancreatic cancer development involves complex mechanisms beyond oxidative stress alone, so the role of antioxidants, including vitamin E, in preventing or treating pancreatic cancer is not fully understood. 

Vitamin C: Vitamin C is a powerful antioxidant that helps protect cells from oxidative stress and damage. It serves as a redox buffer that can lower reactive oxygen species and so neutralize them. Pancreatic cancer development involves complex processes, including DNA damage and inflammation, where antioxidants like vitamin C may play a role [30]. Animal studies have demonstrated that vitamin C can prevent pancreatic preneoplastic lesions. A meta-analysis including 20 observational studies (14 case-control and six cohort) observed almost 5000 patients with pancreatic cancer. Ten of the 14 case-control studies exclusively examined vitamin C in food, two only looked at vitamin C in supplements, and the remaining two examined vitamin C from various sources. Since they contain high levels of dietary vitamin C, fruits and vegetables have long been thought to protect against various malignancies, including pancreatic cancer. A robust inverse association was found in the case-control studies, but as per the authors, the results of these studies might have been influenced by recall and selection biases. More studies are required to analyze the relationship further [31]. Some preclinical studies have explored the potential synergistic effects of combining vitamin C with other treatments, such as chemotherapy or radiation therapy, for pancreatic cancer. These studies have shown mixed results, some suggesting potential benefits while others have not found significant improvements. Although the mechanisms of vitamin C action appear promising, they require strong randomized and controlled clinical trials to be validated [32]. 

Folate: Folate is involved in DNA synthesis and methylation, and adequate levels may play a role in maintaining healthy cell function. One of the main sources of the single-carbon group needed to methylate DNA is folate. Targeted hypermethylation and general hypomethylation are regarded as the defining features of human malignancies [33]. Some studies have suggested a potential association between higher folate intake and a reduced risk of pancreatic cancer. A screening trial investigating the association between dietary folate intake and pancreatic cancer found a statistically significant inverse relationship in women, but no association was found among men [34]. 

Pyridoxine:* *Pyridoxine, also known as vitamin B6, is an essential nutrient that plays a crucial role in various biological processes, including protein metabolism, neurotransmitter synthesis, and immune function [35]. A recent meta-analysis of nine studies showed a significant decrease in the risk of pancreatic cancer with pyridoxine intake [36]. While pyridoxine is an essential nutrient and plays a role in the overall health, including immune function and metabolism, it is not currently recommended as a standalone treatment or preventive measure for pancreatic cancer. 

Cobalamin:* *Vitamin B12 plays a crucial role in methylation, a biochemical process involved in DNA synthesis and repair. Disruptions in DNA repair mechanisms can contribute to cancer development, including pancreatic cancer [37]. A study assessing the relationship between folate, vitamin B6, and vitamin B12 found no association between vitamin B12 and pancreatic cancer [38]. It is important to note that vitamin B12 is essential for numerous bodily functions, including red blood cell production and nerve cell function. 

Minerals

Calcium:* *Numerous biological activities, such as cell division, proliferation, and apoptosis (programmed cell death), depend heavily on calcium. A sufficient calcium level may aid in regulating healthy cell growth and hinder the creation of malignant cells [39]. Additionally, bile acids and fatty acids in the colon can bind to calcium, potentially lessening its adverse effects on the pancreas [40]. Several observational studies have explored the relationship between calcium intake or blood levels and pancreatic cancer risk. Some studies have reported a modest inverse association, suggesting that higher calcium intake or blood levels may be associated with a reduced risk of pancreatic cancer. Recently, the Prostate, Lung, Colorectal, and Ovarian (PLCO) Cancer Screening trial analyzed the relationship between calcium and the risk of pancreatic cancer, but the association was restricted to those with a high fat intake [41]. 

Magnesium:* *Studies show that magnesium has a negative correlation with diabetes risk, which is a risk factor for pancreatic cancer. In the VITamins and Lifestyle (VITAL), a cohort of 66,806 men and women aged 50-76 was analyzed. During an almost seven-year follow-up, 151 participants developed pancreatic cancer. According to the study, a reduction of daily magnesium intake by 100 mg led to a 24% rise in the risk of pancreatic cancer. There was a 76% increase in the risk of pancreatic cancer among individuals who consumed less magnesium than the recommended dietary allowance (RDA) compared to those who consumed the recommended amount. Taking the recommended dose of magnesium can be beneficial in preventing pancreatic cancer [42].

Selenium:* *Selenium is a component of several antioxidant enzymes, including glutathione peroxidase, which helps protect cells from oxidative damage. Oxidative stress is thought to play a role in developing pancreatitis, and selenium's antioxidant properties may contribute to mitigating the damage [43]. In a retrospective cohort study that assessed serum concentrations of selenium and copper in patients diagnosed with pancreatic cancer, low levels of selenium were associated with pancreatic cancer development, and a high level was found to be associated with prolonged survival in pancreatic cancer patients [44].

Zinc:* *Prior studies have hypothesized a possible connection between dietary zinc consumption and the risk of pancreatic cancer. A meta-analysis of two cohort studies and five case-control studies involving 1659 cases was conducted in light of inconsistent data. The study concluded that dietary zinc intake can significantly reduce the risk of pancreatic cancer [45].

Copper:* *Copper's pro-oxidant properties and its role in promoting angiogenesis are thought to potentially contribute to cancer development. In a retrospective cohort study that assessed serum concentrations of selenium and copper in patients diagnosed with pancreatic cancer, high levels of copper might be associated with pancreatic cancer development [44].

Relationship between beverage consumption and pancreatic cancer

Tea

Some observational studies have suggested a potential protective effect of tea, particularly green tea, against pancreatic cancer. These studies have found that regular tea consumption, four glasses daily for at least four months, decreases the amount of 8-hydroxydeoxyguanosine in urine, which may be associated with a reduced risk of pancreatic cancer. Tea contains various bioactive compounds, such as polyphenols (e.g., catechins), which have been studied for their potential anticancer effects. These compounds have been found to exhibit antioxidant, anti-inflammatory, and anticarcinogenic properties in laboratory studies [46,47]. A case-controlled study conducted in China found a connection between consuming green tea and a lower risk of pancreatic cancer. Drinking tea three times a week for more than six months was associated with a reduced likelihood of pancreatic cancer in women. Regardless of the amount or frequency of tea intake, men and women who frequently consume tea have a reduced likelihood of developing pancreatic cancer [48].

Coffee

The association between the consumption of coffee and the risk of pancreatic cancer has been analyzed in several observational studies. Most of these studies have discovered an inverse correlation, indicating a higher coffee intake may be linked to a lower risk of pancreatic cancer. However, the association's strength and the specific amount or type of coffee required for a potential protective impact are yet unknown. A meta-analysis of 14 cohort studies that included 671,080 individuals (1496 cancer events) with an average follow-up of 14.9 years showed an inverse relationship between the risk of pancreatic cancer and consuming coffee. In a subgroup analysis of this study, an inverse association was found among men, but no association was found among women [49]. 

Alcohol

Multiple studies have clearly demonstrated that excessive and persistent alcohol use increases the likelihood of developing pancreatic cancer. The risk appears to be dose-dependent, indicating that it increases as more alcohol is consumed. More than three to four alcoholic beverages per day are considered heavy alcohol intake. Researchers are still uncertain about the specific mechanisms through which alcohol increases the risk of pancreatic cancer. Nevertheless, several putative processes have been proposed. Alcohol may produce oxidative stress, which damages cells and results in genetic changes that can lead to cancer. Alcohol can also directly harm DNA. Additionally, alcohol may increase the synthesis of certain enzymes that turn procarcinogens into carcinogens. Additionally, it is thought that high alcohol intake is a risk factor for pancreatic cancer since it is linked to pancreatitis [50]. A recent dose-response meta-analysis of 19 cohort studies, including 4,211,129 participants, showed that the risk of pancreatic cancer was low to negligible with low to moderate alcohol use. High consumption of alcohol was associated with an increased likelihood of pancreatic cancer [51]. In another Swedish retrospective cohort study, pancreatic cancer risk was analyzed among the patients admitted to the hospital for alcoholism, alcoholic chronic pancreatitis, non-alcoholic chronic pancreatitis, alcoholic liver cirrhosis, or non-alcoholic liver cirrhosis. It showed only a moderate increase in the risk of pancreatic cancer. A moderate increase in risk was found among patients with alcoholic liver cirrhosis, while no significant increase in risk was found among patients with non-alcoholic liver cirrhosis. The increased risk in patients with alcoholic cirrhosis might have been confounded by smoking [52]. When alcohol consumption is combined with smoking, the risk of pancreatic cancer is even higher than when either factor is present alone. It is important to note that moderate alcohol consumption, defined as up to one drink per day for women and up to two drinks per day for men, does not appear to significantly increase the risk of pancreatic cancer [53]. Reducing alcohol consumption, or abstaining from alcohol altogether, is generally recommended to minimize the risk of pancreatic cancer and other alcohol-related health problems.

Dietary patterns and pancreatic cancer risk

Western Diet

According to studies, a Western diet consisting of red and processed meat consumption, refined grains, high-fat dairy products, high-sugar drinks and candy, and a limited intake of fruits and vegetables may raise the risk of pancreatic cancer. A case-controlled study conducted in San Francisco analyzed 532 cases and 1701 controls. The study showed that an increased intake of fruits, vegetables, fish, poultry, whole grains, and low-fat dairy products is indicative of a prudent dietary pattern, which has been linked to a roughly 50% lower risk of pancreatic cancer in males. A higher intake of red and processed meats, potato chips, sugary drinks, sweets, high-fat dairy, eggs, and refined grains are a hallmark of the Western diet, which has been linked to an increased risk of pancreatic cancer in men with an attributed risk of 2.4 but not in women. The risk was tripled for men in the highest quintiles of the Western diet and the lower quintiles of the prudent diet [54]. Consuming meals rich in sugar regularly might increase the likelihood of developing pancreatic cancer by frequently causing postprandial hyperglycemia, raising insulin demands, and lowering insulin sensitivity. In a prospective study that analyzed 131 cases, consumption of soft drinks, fruit soups with added sugar, and sweetened fruit stews were strongly correlated with a higher risk of pancreatic cancer [55].

Mediterranean Dietary Pattern

The Mediterranean diet is characterized by high consumption of fruits, vegetables, whole grains, legumes, nuts, seeds, fish, and olive oil while being low in red and processed meats, saturated fats, and added sugars [56]. Several studies have suggested that adherence to a Mediterranean diet is associated with a reduced risk of pancreatic cancer. A recent meta-analysis of eight studies showed that higher adherence to the Mediterranean diet is associated with a reduced risk of pancreatic cancer. The sensitivity studies show that the relationship does not seem to depend on the particular Mediterranean Diet score utilized or geographic distribution. Nevertheless, women have been found to experience a greater risk reduction than men [57]. Adopting a Mediterranean dietary pattern and other healthy lifestyle choices such as regular physical activity and avoiding smoking may contribute to overall health and potentially lower the risk of pancreatic cancer. 

Healthy Dietary Patterns

A decreased incidence of pancreatic cancer has also been linked to good eating habits, including DASH (Dietary Approaches to Stop Hypertension) and plant-based diets. These eating habits emphasize fresh produce, whole grains, lean proteins, and low-fat dairy products while restricting the intake of processed foods, sugary drinks, and red and processed meats. Consuming various fruits and vegetables, including leafy greens, cruciferous vegetables, berries, and citrus fruits, is associated with a lower risk of pancreatic cancer. Consuming cruciferous, vegetables - especially raw ones - is a modifiable lifestyle habit that may have a negative association with pancreatic cancer [58].

## Conclusions

In conclusion, current epidemiological data demonstrate that nutrition is critical in developing pancreatic cancer despite significant knowledge gaps relating to food and pancreatic cancer risk. Pancreatic cancer risk is higher in overweight women who consume a lot of carbohydrates and have sedentary lifestyles. A high glycemic index and glycemic load diet are linked to a higher risk of developing pancreatic cancer. Plant-based proteins appear to have an inverse link with the risk of pancreatic cancer, but animal proteins show a substantial positive correlation. While polyunsaturated fats and olive oil show an inverse association with pancreatic cancer risk, saturated and monounsaturated fats have a direct correlation. When it comes to micronutrients, a larger intake of copper raises the risk of pancreatic cancer. Still, adequate intake of vitamin D, E, C, B-complex, calcium, selenium, zinc, and magnesium can reduce the risk. Tea and coffee use revealed a decreased risk of pancreatic cancer. Moderate alcohol consumption does not show a connection. However, high drinking is linked to an elevated risk. When comparing different dietary patterns, a Western diet raises the risk since it contains ingredients that increase the risk of pancreatic cancer. However, a Mediterranean or healthy balanced diet, such as the DASH diet, lowers the risk. More studies are warranted to study the effects of diet and dietary patterns on the risk of pancreatic cancer.
